# Effectiveness of Digital Health Literacy Interventions in Older Adults: Single-Arm Meta-Analysis

**DOI:** 10.2196/48166

**Published:** 2023-06-28

**Authors:** Qian Dong, Ting Liu, Ran Liu, Hongxia Yang, Cuiping Liu

**Affiliations:** 1 School of Nursing Shandong First Medical University & Shandong Academy of Medical Sciences Taian, Shandong Province China; 2 School of Nursing Qingdao University Qingdao, Shandong Province China; 3 Jinan Blood Center Jinan, Shandong Province China

**Keywords:** digital health literacy interventions, older adults, eHealth literacy efficacy, knowledge, self-efficacy, skills

## Abstract

**Background:**

In a world of rapid digital technology development, the lack of digital health literacy (DHL) among older people cannot be ignored. DHL is becoming an essential competency that can facilitate the health status and health management of older adults. DHL interventions that are feasible and appropriate can be implemented on a large scale through the health care system for older people.

**Objective:**

The purpose of this meta-analysis was to assess the effectiveness of DHL interventions for older adults.

**Methods:**

English publications in PubMed, Web of Science, Embase, and the Cochrane Library were searched from inception to November 20, 2022. Two reviewers independently completed the data extraction and quality assessment. Review Manager (version 5.4; Cochrane Informatics & Technology Services) software was used for all meta-analyses.

**Results:**

A total of 7 studies, including 2 randomized controlled trials and 5 quasi-experimental studies, involving 710 older adults were considered eligible. The main outcome was scores on the eHealth Literacy Scale, and secondary outcomes were knowledge, self-efficacy, and skills. Quasi-experimental studies compared baseline and postintervention outcomes, while randomized controlled trials compared pre- and postintervention outcomes in the intervention group. Of the 7 studies, 3 used face-to-face instruction, while 4 adopted web-based interventions. Among them, 4 of the interventions were conducted using theoretical guidance, while 3 were not. Intervention duration varied from 2 to 8 weeks. In addition, the studies included were all conducted in developed countries, mainly in the United States. Pooled analysis presented that DHL interventions had positive effects on eHealth literacy efficacy (standardized mean difference 1.15, 95% CI 0.46 to 1.84; *P*=.001). Subgroup analysis revealed that DHL interventions that chose face-to-face teaching (standardized mean difference 1.15, 95% CI 0.46 to 1.84; *P*=.001), were guided by a conceptual framework (standardized mean difference 1.15, 95% CI 0.46 to 1.84; *P*=.001), and were sustained over 4 weeks (standardized mean difference 1.1, 95% CI 0.46 to 1.84; *P*=.001) had a more significant effect. Moreover, the outcomes showed considerable gains in knowledge (standardized mean difference 0.93, 95% CI 0.54 to 1.31; *P*<.001) and self-efficacy (standardized mean difference 0.96, 95% CI 0.16 to 1.77; *P*=.02). No statistically significant effect was found for skills (standardized mean difference 0.77, 95% CI –0.30 to 1.85; *P*=.16). The small number of studies, variable study quality, and heterogeneity are some limitations of this review.

**Conclusions:**

DHL interventions have positive effects on the health status and health management of older adults. Practical and effective DHL interventions are crucial for the use of modern digital information technology in managing the health of older people.

**Trial Registration:**

PROSPERO International Prospective Register of Systematic Reviews CRD42023410204; https://www.crd.york.ac.uk/prospero/display_record.php?RecordID=410204

## Introduction

Digital health literacy (DHL) is an extension of eHealth literacy [1], which refers to the ability of individuals as active participants who can search for, understand, analyze, and evaluate health information on the internet or in other digital sources and share the information with others to help make health-related decisions and solve health problems, both individually and collectively [2]. DHL stresses the individual as a participant and sharer, not just a recipient. However, eHealth literacy only emphasizes the individual as a mere receiver. Some studies have shown that older adults with a higher DHL may have more health knowledge and more appropriate attitudes toward medical decision-making [3,4].

Several factors are likely to be associated with the DHL of an individual, including computer or internet usage (number of electronic devices, frequency of internet use, and computer stress), age, income, education, personal experiences, marital status, and social support [3,5]. Unlike young people born in the digital age, older people may have less experience in using modern media technologies and platforms [6], while many older individuals find it difficult to use digital media, which they deem to be complicated and sophisticated [7].

In recent years, along with the increase in the aging population, older adults with multiple chronic diseases and physical disabilities who are living alone have become an increasingly large group [8-11]. Meanwhile, advancements in science and technology have made a dramatic difference in personal medical experiences, health self-management, and social health services. At present, telemedicine and tele-homecare are emerging as a rapidly growing segment of the health care industry that can provide assistance and care to older adults [12,13]. As a group with a high prevalence of difficult-to-treat illnesses and conditions, older adults have a greater need for telehealth [14]. For example, interventions for tele–transition of care can reduce readmission and mortality rates and improve health-related quality of life for older adults at high risk of readmission [15]. In addition, the internet has become one of the main sources of health information. The types of web-based health information searched by older people mainly include diseases, treatment, nutrition, and exercise [16]. However, some older people find it difficult to use digital health care services due to a lack of ability to search for web-based health information and low DHL [17]. Therefore, older people should be aware of the importance of seeking health information on the internet and improving their DHL. Older adults with a high DHL are able to accurately search for and discriminate health information, judge the veracity of the context, and improve resilience to misinformation on the internet [18]. Moreover, the improvements in DHL could facilitate medical procedures, personal health management skills, and life satisfaction of individuals [19].

Practical and effective DHL interventions are crucial for improving DHL and the ability to use information technology among older adults [20,21]. Some DHL interventions, such as training on computer knowledge, attitudes, and skills, may be the key to improving the understanding and application of eHealth among older adults [22-24]. The target audience for these interventions are mostly community-dwelling older adults. The contents of the intervention include the following main areas: computer basics, mainstream search engines, official patient portals, and peer support forums. The methods of interventions generally involve digital and on-site education or training [25]. However, current DHL interventions vary widely. For example, intervention periods range from 2 weeks to 8 weeks or even longer [24]. The intervention groups are either older individuals in the community or hospital patients [26].

Due to their variability, the effectiveness of DHL interventions needs to be further explored. Therefore, this review aims to systematically summarize the effectiveness of DHL interventions in older adults in terms of eHealth literacy efficacy, knowledge, self-efficacy, and skills.

It is expected that this review will provide a practical basis and theoretical support for future research and the formulation of DHL interventions for older adults.

## Methods

### Overview

This review followed the PRISMA (Preferred Reporting Items for Systematic Reviews and Meta-Analyses) guidelines published in 2020 [27] and was registered in PROSPERO (CRD42023410204).

### Search Strategy

We searched PubMed, Web of Science, Embase, and the Cochrane Library for all available relevant studies published from inception until November 20, 2022. The search strategy used Boolean logic to combine medical subject headings and text word searches. We also screened the references of relevant prior systematic reviews as additional sources, and the language was limited to English. The complete search strategy can be found in Multimedia Appendix 1. An example of the search strategy used on the PubMed database is as follows:

((“Computer Literacy”[Mesh]) OR ((((((((((((Computer Literacy[Title/Abstract]) OR (Computer Literacies[Title/Abstract])) OR (Literacies, Computer[Title/Abstract])) OR (Literacy, Computer[Title/Abstract])) OR (ehealth literacy[Title/Abstract])) OR (e-health literacy[Title/Abstract])) OR (digital literacy[Title/Abstract])) OR (digital health literacy[Title/Abstract])) OR (mhealth literacy[Title/Abstract])) OR (m-health literacy[Title/Abstract])) OR (telehealth literacy[Title/Abstract])) OR (tele-health literacy[Title/Abstract]))) AND ((((((aged[Title/Abstract]) OR (elderly[Title/Abstract])) OR (old adult[Title/Abstract])) OR (older adult[Title/Abstract])) OR (old people[Title/Abstract])) OR (older people[Title/Abstract]))

### Eligibility Criteria

Eligibility criteria were formulated based on the PICOS framework (populations, interventions, comparisons, outcomes, and study design): (1) populations—participants were older adults (average age 60 years or older); (2) interventions—any form of eHealth literacy and DHL interventions for older individuals (eg, web-based courses and face-to-face teaching); (3) comparisons—preintervention; (4) outcomes—main outcome was eHealth literacy efficacy (the 8-item eHealth Literacy Scale [e-HEALS] [28] scores, mean, or total scores) and secondary outcomes were knowledge, self-efficacy, and skills; the study included at least 1 secondary indicator to assess DHL, in addition to eHealth literacy efficacy as the main outcome; (5) study design—randomized controlled trials (RCTs) or quasi-experimental studies (single-group pretest-posttest design).

Moreover, the e-HEALS was presented in Multimedia Appendix 2.

Exclusion criteria included (1) nonexperimental trials, (2) review or discussion papers, (3) non-English papers, (4) studies with full text unavailable, and (5) studies with insufficient data.

### Study Selection

Two reviewers (QD and RL) independently performed the screening and selection of studies. Disagreements were resolved through discussion or consultation with a third reviewer (TL). Duplicates were first removed, and then the title and abstract were assessed for eligibility. In addition, clearly incompatible studies were excluded after the title and abstract were screened. Finally, full-text and the identifications of qualified studies that strictly adhered to the inclusion and exclusion criteria [29] were screened.

### Data Extraction

Data extraction from the studies included was performed by 2 independent reviewers. Characteristics of the studies (authors, country, study design, and sample characteristics) and intervention characteristics (intervention methods, theory framework, duration, and results) were included.

### Quality Appraisal

The Joanna Briggs Institute quality measurement was used to assess the risk of bias in each trial included. Nine aspects (eg, causality and baseline comparability) of the risk of bias were assessed in the quasi-experimental studies. Thirteen aspects (eg, randomization, allocation concealment, baseline, researcher blindness, and outcome assessors) were evaluated for the risk of bias in the RCT studies. Each item could be answered as “yes,” “no,” “unclear,” or “inapplicable.” In the event of disagreement, a third reviewer was requested to arbitrate.

### Statistical Analysis

In this meta-analysis, the quasi-experimental trials compared baseline and postintervention outcomes, while the RCTs compared pre- and postintervention outcomes in the intervention group.

Based on the Cochrane Handbook for Systematic Reviews of Interventions, heterogeneity was assessed using the *I*^2^ statistic, which ranges from 0 to 100 (75%-100%=large, 50%-90% =substantial, 30%-60%=moderate, 0%-40%=inessential) [30]. Since the continuous outcomes were assessed by different scales, we used the standardized mean difference (SMD) and expressed the results as 95% CIs. We chose a random effects model due to the high level of heterogeneity among the studies included as a result of differences in the study design, intervention approaches, and data collection methods. All meta-analyses were performed using ReviewManager (version 5.4; Cochrane Collaboration), and statistical significance was defined as a *P* value of <.05. It is possible that the final pooled results show considerable heterogeneity, and we performed a sensitivity analysis to identify the cause of a high degree of heterogeneity. Subgroup analyses of the study groups were performed for intervention methods, theoretical guidelines, and duration to assess the components that contributed to the intervention effects.

## Results

### Identification of Studies

Initially, 3145 studies were retrieved from the selected databases, and after the removal of duplicates, 2799 studies remained.

**Figure 1:**
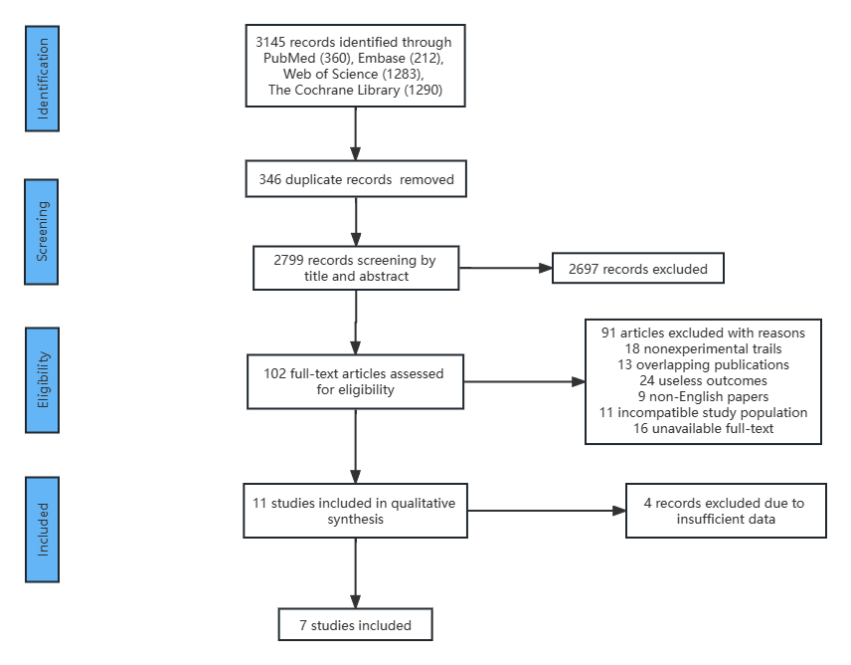
PRISMA (Preferred Reporting Items for Systematic Reviews and Meta-Analyses) flow diagram: papers included and excluded in this review.

After the titles and abstracts were screened, only 102 studies were included in the full-text screening process. Subsequently, 91 studies were excluded as they did not meet the eligibility criteria. The detailed reasons for exclusion are as follows: 18 were nonexperimental trials, 13 were overlapping publications, 24 provided results that were useless, 9 were non-English reports, 11 included incompatible study populations, and the full text was unavailable for 16 publications. Additionally, 4 trials were excluded due to inadequate outcome data. Finally, 7 trials were included in this meta-analysis. The study screening process and the results are shown in Figure 1.

### Characteristics of Included Studies

This meta-analysis included 7 trials conducted on 710 older individuals with an average age of 60 years or older. Two of these trials were RCTs conducted on 187 older adults in the experimental groups and 184 in the control groups. Five quasi-experimental trials used a single-group pretest-posttest design to develop and evaluate DHL interventions for older people. Moreover, the trials included were implemented across different countries, predominantly the United States. The characteristics of included studies for this meta-analysis are shown in Multimedia Appendix 3 [31-37]. The raw data sets for this study are presented in Multimedia Appendix 4.

### Quality of Assessment

Tables 1 and 2 show the results of the quality assessment of the studies included.

For the quasi-experimental trials, 4 trials were rated A (high quality), while 1 was rated B (moderate quality). Clear cause and effect, control group, the same outcome measurement, reliability of outcome measurements, and appropriate statistical analysis were reported in all quasi-experimental trials. However, none of these trials reported similarity at the baseline, while 4 trials did not report whether the groups received the same treatment as the 1 being validated and 2 trials did not provide information on follow-up. One trial was rated B (moderate quality) because multiple measurements were not performed. The 2 RCTs were rated B because they did not contain true randomization, allocation concealment, and blinding of subjects and researchers.

**Table 1:** Risk of bias assessment for the quasi-experimental trials.

Reference	Q1^a^	Q2^b^	Q3^c^	Q4^d^	Q5^e^	Q6^f^	Q7^g^	Q8^h^	Q9^i^	Quality grade
Xie (2011) [31]	Y^j^	U^k^	U	Y	Y	Y	Y	Y	Y	A
Chiu et al (2016) [33]	Y	U	Y	Y	Y	Y	Y	Y	Y	A
Lee and Kim (2019) [32]	Y	U	U	Y	Y	U	Y	Y	Y	A
Bevilacqua et al (2021) [34]	Y	U	U	Y	Y	Y	Y	Y	Y	A
Chang et al (2021) [35]	Y	U	U	Y	N^l^	U	Y	Y	Y	B

^a^Q1: clear cause and effect.

^b^Q2: similar at baseline.

^c^Q3: similar treatment other than the intervention.

^d^Q4: control group.

^e^Q5: multiple measurements.

^f^Q6: follow-up description.

^g^Q7: the same outcome measurement.

^h^Q8: reliability of outcome measurements.

^i^Q9: appropriate statistical analysis.

^j^Y: yes.

^k^U: unclear.

^l^N: no.

**Table 2:** Risk of bias for the randomized controlled trials.

Reference	Q1^a^	Q2^b^	Q3^c^	Q4^d^	Q5^e^	Q6^f^	Q7^g^	Q8^h^	Q9^i^	Q10^j^	Q11^k^	Q12^l^	Q13^m^	Quality grade
Nahm et al (2019) [36]	U^n^	U	Y^o^	U	U	Y	Y	Y	Y	Y	Y	Y	Y	B
De Main et al (2022) [37]	U	U	Y	Y	Y	Y	Y	Y	Y	Y	Y	Y	Y	B

^a^Q1: true randomization.

^b^Q2: allocation concealment.

^c^Q3: similar at baseline.

^d^Q4: blinding of subjects.

^e^Q5: blinding of the researcher.

^f^Q6: blinding of outcome assessors.

^g^Q7: identical treatment other than the intervention.

^h^Q8: follow-up description.

^i^Q9: intention-to-analysis.

^j^Q10: the same outcome measurement.

^k^Q11: reliability of outcome measurements.

^l^Q12: appropriate statistical analysis.

^m^Q13: appropriate trial design.

^n^Y: yes.

^o^U: unclear.

### Digital Health Literacy Interventions

Of the studies included, 3 entailed face-to-face teaching, while 4 adopted web-based interventions. The face-to-face teaching interventions combined with theoretical knowledge and practical skills were guided by university students and conducted in small groups. Web-based education interventions adopted an innovative training project that provided older adults with DHL to strengthen their health self-management. In addition, 4 interventions were theoretically based, while 3 were not. The duration of 4 of the interventions was more than 4 weeks, while the other 3 were less than 4 weeks long.

### Effect of Digital Health Literacy Intervention

This review systematically assessed the impact of DHL interventions in older adults, using e-HEALS scores as the main measure and also analyzed changes in computer or web knowledge, computer self-efficacy and procedural skills for computer or internet use in the studies included. Subgroup analysis was performed on the eHealth literacy efficacy of the intervention methods, theoretical guidelines, and duration of the intervention.

### eHealth Literacy Efficacy

**Figure 2:**
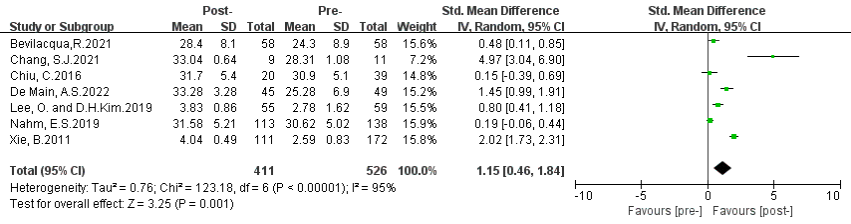
Effects of digital health literacy interventions on eHealth literacy efficacy of older adults [31-37]. IV: inverse variance. Std: standardized.

All the included studies reported a change in the eHealth literacy efficacy of the participants from baseline to post-intervention using an e-HEALS. Two studies [31,33] provided eHealth literacy efficacy in terms of mean scores, while the remaining trials [32,34-37] adopted a total points system. As presented in Figure 2, a significant positive effect was shown on the eHealth literacy efficacy (SMD 1.15, 95% CI 0.46-1.84; *P*=.001). We speculate that the considerable degree of heterogeneity (*P*<.001, *I*^2^=95%) could be explained by the different forms of data collection, intervention methods, and duration of the interventions, as well as the presence or absence of theoretical guidance.

### Subgroup Analysis

#### Overview

**Figure 3:**
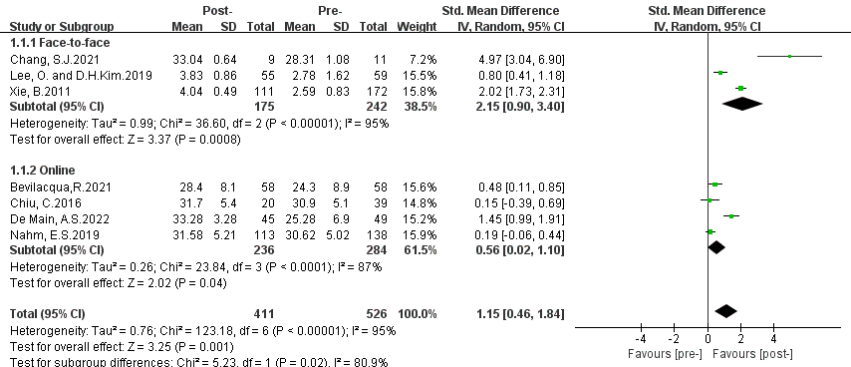
Subgroup analysis on the outcome of eHealth literacy efficacy categorized by intervention methods (group 1: face-to-face teaching; group 2: web-based education) [31-37]. IV: inverse variance. Std: standardized.

**Figure 4:**
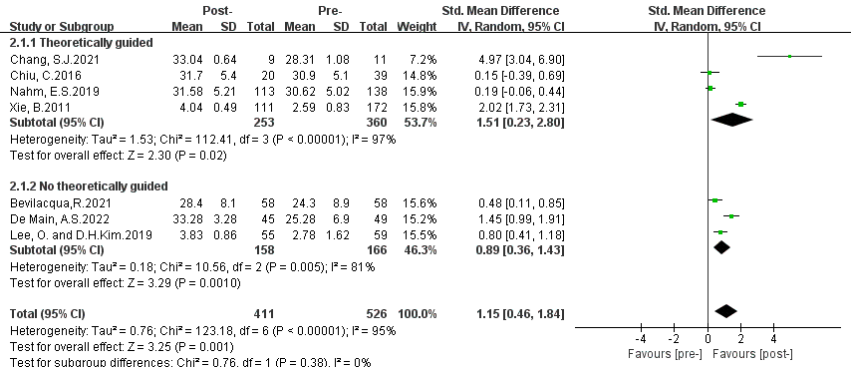
Subgroup analysis on the outcome of eHealth literacy efficacy categorized by theoretical guidelines (group 1: theoretically guided interventions; group 2: no theoretically guided interventions) [31-37]. IV: inverse variance. Std: standardized.

**Figure 5:**
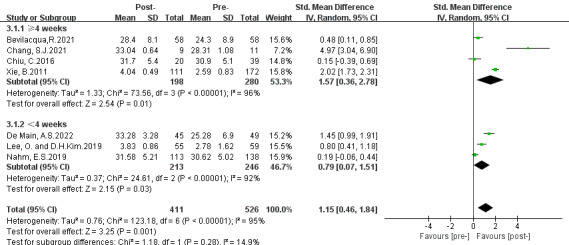
Subgroup analysis on the outcome of eHealth literacy efficacy categorized by length of whole duration (group 1: duration ≥4 weeks; group 2: duration <4 weeks) [31-37]. IV: inverse variance. Std: standardized.

Three subgroup analyses were conducted based on intervention approaches, theoretical guidelines, and intervention duration. The results are presented in Figures 3-5.

#### First Subgroup Categorized by Intervention Methods

The first subgroup analysis was conducted based on the intervention approaches, including (1) face-to-face and (2) on the internet. Subgroup 1 results presented a greater positive effect on eHealth literacy efficacy (SMD 2.15, 95% CI 0.90-3.40; *P*<.001), and subgroup 2 revealed a moderate effect (SMD 0.56, 95% CI 0.02-1.10; *P*=.04). Both groups showed a considerable level of heterogeneity (group 1: *P*<.001, *I*^2^=95%; group 2: *P*<.001, *I*^2^=87%). Subgroup differences were observed (*P*=.02, *I*^2^=80.9%). Moreover, a positive overall significance was discovered (SMD 1.15, 95% CI 0.46-1.84; *P*=.001) in favor of DHL interventions.

#### Second Subgroup Categorized by Theoretical Guidelines

The second subgroup analysis was performed based on the theoretical guidelines, including (1) theoretically guided and (2) no theoretical guidance. The results of subgroups 1 and 2 showed greater effectiveness in improving eHealth literacy efficacy (subgroup 1: SMD 1.51, 95% CI 0.23-2.80; *P*=.02; subgroup 2: SMD 0.89, 95% CI 0.36-1.43; *P*=.001). Both subgroups presented a high degree of heterogeneity (group 1: *P*<.001, *I*^2^=97%; group 2: *P*=.005, *I*^2^=81%). Subgroup differences were observed (*P*=.38, *I*^2^=0%). Moreover, a positive effect was found (SMD 1.15, 95% CI 0.46-1.84; *P*=.001) in favor of DHL interventions.

#### Third Subgroup Categorized by the Length of Whole Intervention Duration

The third subgroup analysis was performed based on intervention duration and included (1) duration ≥4 weeks and (2) duration <4 weeks. The results of subgroup 1 revealed a large significant effect in increasing eHealth literacy efficacy (SMD 1.57, 95% CI 0.36-2.78; *P*=.01) and subgroup 2 presented a moderate statistical significance (SMD 0.79, 95% CI 0.07-1.51; *P*=.03). Both groups presented a high degree of heterogeneity (group 1: *P*<.001, *I*^2^=96%; group 2: *P*<.001, *I*^2^=92%). Subgroup differences were observed (*P*=.28, *I*^2^=14.9%). Moreover, a positive statistical overall effect was shown (SMD 1.15, 95% CI 0.46-1.84; *P*=.001) in favor of DHL interventions.

### Knowledge

**Figure 6:**
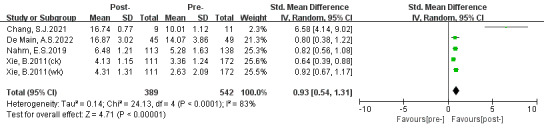
Effects of digital health literacy interventions on the outcome of knowledge among older adults (before sensitivity analysis) [31,35-37]. IV: inverse variance. Std: standardized.

**Figure 7:**
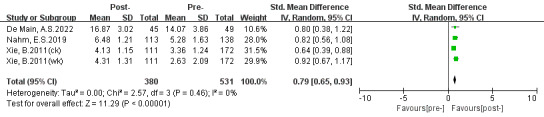
Effects of digital health literacy interventions on the outcome of knowledge among older adults (after sensitivity analysis) [31,36,37]. IV: inverse variance. Std: standardized.

Four studies assessed knowledge of computers, the web, and patient portals. Two studies [35,37] used a questionnaire designed by Xie [38], 1 [36] used a questionnaire used in prior studies [39], and another [31] used objective tests. A statistically considerable effect for knowledge (SMD 0.93, 95% CI 0.54-1.31; *P*<.001) is shown in Figure 6. Sensitivity analysis detected that 1 study [35] was most likely the cause of the high heterogeneity (*P*<.001, *I*^2^=83%). After removing this study, the heterogeneity was low (*P*=.46, *I*^2^=0%) as shown in Figure 7. We thought that the high heterogeneity might be due to differences in the number of participants and questionnaires.

### Self-Efficacy

**Figure 8:**
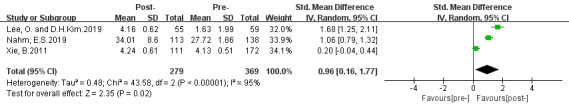
Effects of digital health literacy interventions on the outcome of self-efficacy among older adults [31,32,36]. IV: inverse variance. Std: standardized.

Three trials mentioned self-efficacy on the computer and patient portal. Two studies [31,32] assessed computer efficacy with the 5-item computer efficacy subscale of the Attitudes Toward Computers Questionnaire [40], while 1 study [36] measured patient portals SE using a modified 4-item Self-Efficacy for Computer-Based Personal Health Record scale using the Personal Health Record Self-Efficacy tool [41]. A positive significant effect (SMD 0.96, 95% CI 0.16-1.77; *P*=.02) is presented in Figure 8. The high degree of heterogeneity (*P*<.001, *I*^2^=95%) could be due to the differences in intervention methods and measurement scales. No sources of heterogeneity were discovered in the sensitivity analysis.

### Skills

**Figure 9:**
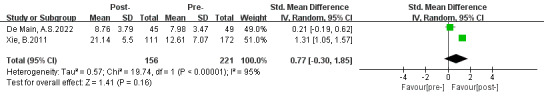
Effects of digital health literacy interventions on the outcome of skills among older adults [31,37]. IV: inverse variance. Std: standardized.

Among the studies included, only 2 studies assessed skills. One study [31] measured skills using a 30-item procedural test and the other [37] assessed skills using a 12-item procedural task. In Figure 9, the pooled effect for skills was not statistically significant (SMD 0.77, 95% CI −0.30 to 1.85; *P*=.16). We thought that the considerable degree of heterogeneity (*P*<.001, *I*^2^=95%) was due to different intervention designs and measurement tests.

## Discussion

### Principal Findings

This meta-analysis evaluated the effects of DHL interventions in 710 older adults. The results showed that DHL interventions had a dramatic impact on eHealth literacy efficacy, knowledge, and self-efficacy, but had no effect on skills.

Although a majority of the studies included were of a quasi-experimental design, the large heterogeneity among the studies could be associated with differences in study subjects, methods, duration of the intervention, and outcome assessment tools. The results demonstrate that the effectiveness of DHL interventions for older adults is convincing.

### Effect of DHL Interventions on eHealth Literacy Efficacy

This meta-analysis revealed that DHL interventions could be an effective way by which older adults could improve their DHL and health self-management. These observations were more evident in the DHL intervention groups that chose appropriate intervention methods, were guided by a conceptual framework, and were sustained for a longer duration.

DHL interventions were delivered through face-to-face teaching or web-based education. Each intervention has its own strengths. During the training process, face-to-face teaching allowed more attention to be paid to physical and psychological changes in the subjects and for adjustments to better meet their needs. This type of training method is usually conducted through group teaching, with 3 to 5 older adults in a group; this can help to promote emotional communication among older people and is popular among this age group. Web-based teaching can break down time and space constraints, giving older people more opportunities to practice independently. Additionally, with web-based teaching, participants can practice the steps they are not proficient in and repeat them as needed. Our meta-analysis showed that face-to-face teaching was more effective than web-based teaching. For instance, a previous study also found that face-to-face interventions were more effective than web-based interventions for psychological interventions [42]. During web-based interventions, there were often many variations that were difficult to address immediately. However, a previous review found that computer-based applications are the most common intervention method [43]. Although web-based courses are becoming more widespread, we still need to consider some obstacles that they present for older adults (for example, they may not have electronic devices, have limited independent learning, and experience computer-related fatigue). Therefore, we should choose appropriate training methods based on the specific conditions of older people who are receiving the training.

In the absence of a unified conceptual framework, DHL interventions often use self-efficacy theory and the Information-Motivation-Behavioral Skills model as theoretical guides. Among the studies included, 2 studies used the self-efficacy theory [31,36], 1 study was guided by the Information-Motivation-Behavioral skills model [35], and another study applied the Technology Acceptance Model and the Diffusion of Innovations model [33]. Self-efficacy theory is defined as the beliefs people hold about their ability to achieve goals in certain domains [44]. The Information-Motivation -Behavioral skills model promotes individuals’ healthy behavior change by giving individuals information, increasing motivation, and providing skills. When individuals have a certain level of information, motivation, and behavioral skills, their behavior changes [45]. The Technology Acceptance Model is a theoretical model that explains users’ acceptance of new technologies and systems [46]. The Diffusion of Innovations model is about persuading people to accept new products, concepts, and things through the media, focusing on the impact of mass communication on society and culture [47]. Compared to other conceptual frameworks, our findings prefer to support the self-efficacy theory to develop the DHL interventions. A previous systematic review also argued that self-efficacy theory was the most appropriate conceptual framework [48]. Theory-based DHL interventions are more systematic and scientific. In this review, DHL interventions based on theoretical frameworks showed outstanding outcomes [36]. Our findings support the results of previous studies that have considered the application of theoretical frameworks in the context of eHealth literacy interventions, indicating that they can have a dramatic impact on intervention effectiveness [48].

DHL interventions typically take around a month and can be as short as 2 weeks or go on for as long as 3 months. A longer duration of intervention may be associated with a better outcome. A previous study supported this perspective by showing that participants’ knowledge and use of digital media increased significantly over a 3-month intervention [49]. In this meta-analysis, DHL interventions that lasted for more than 4 weeks had a better effect than those that lasted for less than 4 weeks. Currently, DHL interventions are typically delivered at a frequency of 2 to 3 times per week. Participants are trained more frequently during longer duration DHL interventions, and therefore these interventions result in a more significant increase in eHealth literacy efficacy.

### Effect of DHL Interventions on Knowledge

Our meta-analysis results revealed that DHL interventions could have a significant difference on the knowledge of older adults. Among the studies included, Nahm et al [36] implemented a theory-based patient portal e-learning program that included 3 modules, with module 1 providing an overview of patient portals (eg, electronic health record and personal health record, patient portal, privacy, and security). Participants in the theory-based patient portal e-learning program intervention group had a clear perception of the project overview in the first phase of the intervention process and therefore improved their patient portals knowledge to better manage their health. A previous trial also adopted this approach and achieved the same results [50]. Armed with a wealth of digital health knowledge, older adults could become empowered to access health information for better alternative decision-making [51]. As a result, it will be possible for older adults to identify health problems and use web-based information to address them.

### Effect of DHL Interventions on Self-Efficacy

Self-efficacy refers to a personal belief in one’s ability to perform the behaviors necessary to achieve a specific performance [44]. In this review, self-efficacy refers to an individual’s computer and internet use–related beliefs and skills. Our findings show that the DHL interventions had a statistical effect on self-efficacy. One of the factors preventing individuals from using the internet is a lack of computer self-efficacy [52,53]. DHL interventions have been proven to have a positive effect on computer self-efficacy in older adults, as shown in this review. Furthermore, scholars have found that people with a higher level of web-based self-efficacy had a higher DHL [54]. In an RCT, older people aged ≥60 years were trained on information and communication technologies, such as email and the internet [49]. The results showed that the experimental group with greater computer self-efficacy was more comfortable in performing computer-related tasks and had a significantly better quality of life than the control group; this is similar to the findings of this review.

### Effect of DHL Interventions on Skills

In terms of improving the skills of older adults, the DHL interventions showed no statistical impact. This result can be explained by the trial by De Main et al [37]. The authors reported that participants in the intervention group who were provided with a multimedia-based tutorial, Online Tutorial Overlay Presenter, may have experienced computer-related fatigue. In addition, according to the Knowledge-Attitude -Practice Model, behavioral change is the most difficult to achieve [55]. In this review, the duration of each intervention was relatively short, and it may be difficult for older adults to improve their procedural skills related to computer and internet use in such a short period of time. A previous mixed methods study reported that experimental participants in a web-based health education program had significantly higher internet use skills [56]. This may be due to the fact that participants in this trial were younger (mean age 50 years) and more experienced with the internet than participants in the studies included in our meta-analysis. We hope that future DHL interventions will be more effective in improving skills in discerning the veracity of web-based information and in operating computers and the internet.

### Limitations

The limitations of the review must be acknowledged. The studies included were all published in English, which may have introduced a language bias. Considerable heterogeneity was attributed to differences in study populations, intervention duration, training methods, and data collection. Furthermore, the number of studies included was small, and therefore the use of funnel plots to assess publication bias was greatly restricted, as there may have been some publication bias. Finally, all of the trials included were conducted in developed countries and regions, which may also cause bias in the results.

### Conclusions

DHL interventions benefit the health status and health management of older adults. This meta-analysis reports on the effectiveness of DHL interventions conducted in developed countries. It shows a positive effect on eHealth literacy efficacy, knowledge, and self-efficacy but no effect on skills. However, the existing interventions did not take into account certain influencing factors, such as the current state, health literacy levels, changes in older adults, and their environment during the intervention process. In addition, there is a lack of a unifying conceptual framework that can guide the development of DHL intervention strategies for older individuals. Future studies should focus on developing interventions that are more feasible and responsive to the real needs of older adults in the digital age for widespread implementation in health care for older people.

## Appendix

Multimedia Appendix 1 The complete search strategy of the meta-analysis.

Multimedia Appendix 2 e-HEALS: The eHealth Literacy Scale.

Multimedia Appendix 3 Characteristics of studies included in the meta-analysis (7 studies).

Multimedia Appendix 4 Data sets.

## Abbreviations

DHL: digital health literacy
e-HEALS: eHealth Literacy Scale
PICOS: populations, interventions, comparisons, outcomes, and study design
PRISMA: Preferred Reporting Items for Systematic Reviews and Meta-Analyses
RCT: randomized controlled trial
SMD: standardized mean difference

## Data Availability

All data generated or analyzed during this study are included in this published article and its supplementary information files.

## References

[1] Norman CD, Skinner HA (2006). eHealth literacy: essential skills for consumer health in a networked world. J Med Internet Res.

[2] Bittlingmayer UH, Dadaczynski K, Sahrai D, van den Broucke S, Okan O (2020). [Digital health literacy-conceptual contextualization, measurement, and promotion]. Bundesgesundheitsblatt Gesundheitsforschung Gesundheitsschutz.

[3] Arcury TA, Sandberg JC, Melius KP, Quandt SA, Leng X, Latulipe C, Miller DP, Smith DA, Bertoni AG (2020). Older adult internet use and eHealth literacy. J Appl Gerontol.

[4] Battineni G, Baldoni S, Chintalapudi N, Sagaro GG, Pallotta G, Nittari G, Amenta F (2020). Factors affecting the quality and reliability of online health information. Digit Health.

[5] Hoogland AI, Mansfield J, Lafranchise EA, Bulls HW, Johnstone PA, Jim HSL (2020). eHealth literacy in older adults with cancer. J Geriatr Oncol.

[6] Song Y, Qian C, Pickard S (2021). Age-related digital divide during the COVID-19 pandemic in China. Int J Environ Res Public Health.

[7] Nicosia J, Aschenbrenner AJ, Adams SL, Tahan M, Stout SH, Wilks H, Balls-Berry JE, Morris JC, Hassenstab J (2022). Bridging the technological divide: stigmas and challenges with technology in digital brain health studies of older adults. Front Digit Health.

[8] McGilton KS, Vellani S, Yeung L, Chishtie J, Commisso E, Ploeg J, Andrew MK, Ayala AP, Gray M, Morgan D, Chow AF, Parrott E, Stephens D, Hale L, Keatings M, Walker J, Wodchis WP, Dubé V, McElhaney J, Puts M (2018). Identifying and understanding the health and social care needs of older adults with multiple chronic conditions and their caregivers: a scoping review. BMC Geriatr.

[9] Kojima G (2017). Frailty as a predictor of disabilities among community-dwelling older people: a systematic review and meta-analysis. Disabil Rehabil.

[10] Mitzner TL, Chen TL, Kemp CC, Rogers WA (2014). Identifying the potential for robotics to assist older adults in different living environments. Int J Soc Robot.

[11] Berridge C (2018). Medicaid becomes the first third-party payer to cover passive remote monitoring for home care: policy analysis. J Med Internet Res.

[12] Liang HY, Lin LH, Chang CY, Wu FM, Yu S (2021). Effectiveness of a nurse-led tele-homecare program for patients with multiple chronic illnesses and a high risk for readmission: a randomized controlled trial. J Nurs Scholarsh.

[13] Weinstein RS, Krupinski EA, Doarn CR (2018). Clinical examination component of telemedicine, telehealth, mHealth, and connected health medical practices. Med Clin North Am.

[14] Biese K, Handler SM, Wardlow L, Agha Z (2022). Telehealth with older adults: getting it right. J Am Geriatr Soc.

[15] Soh YY, Zhang H, Toh JJY, Li X, Wu XV (2023). The effectiveness of tele-transitions of care interventions in high-risk older adults: a systematic review and meta-analysis. Int J Nurs Stud.

[16] Zhao YC, Zhao M, Song S (2022). Online health information seeking behaviors among older adults: systematic scoping review. J Med Internet Res.

[17] Yao R, Zhang W, Evans R, Cao G, Rui T, Shen L (2022). Inequities in health care services caused by the adoption of digital health technologies: scoping review. J Med Internet Res.

[18] Moore RC, Hancock JT (2022). A digital media literacy intervention for older adults improves resilience to fake news. Sci Rep.

[19] Choukou MA, Sanchez-Ramirez DC, Pol M, Uddin M, Monnin C, Syed-Abdul S (2022). COVID-19 infodemic and digital health literacy in vulnerable populations: a scoping review. Digit Health.

[20] Cotten SR (2017). Examining the roles of technology in aging and quality of life. J Gerontol B Psychol Sci Soc Sci.

[21] Chang SJ, Jang SJ, Lee H, Kim H (2021). Building on evidence to improve ehealth literacy in older adults: a systematic review. Comput Inform Nurs.

[22] Creber RMM, Hickey KT, Maurer MS (2016). Gerontechnologies for older patients with heart failure: what is the role of smartphones, tablets, and remote monitoring devices in improving symptom monitoring and self-care management?. Curr Cardiovasc Risk Rep.

[23] Arcury TA, Quandt SA, Sandberg JC, Miller DP, Latulipe C, Leng X, Talton JW, Melius KP, Smith A, Bertoni AG (2017). Patient portal utilization among ethnically diverse low income older adults: observational study. JMIR Med Inform.

[24] Lin YH, Lou MF (2021). Effects of mHealth-based interventions on health literacy and related factors: a systematic review. J Nurs Manag.

[25] Wang X, Luan W (2022). Research progress on digital health literacy of older adults: a scoping review. Front Public Health.

[26] Axelrod DA, Kynard-Amerson CS, Wojciechowski D, Jacobs M, Lentine KL, Schnitzler M, Peipert JD, Waterman AD (2017). Cultural competency of a mobile, customized patient education tool for improving potential kidney transplant recipients' knowledge and decision-making. Clin Transplant.

[27] Page MJ, McKenzie JE, Bossuyt PM, Boutron I, Hoffmann TC, Mulrow CD, Shamseer L, Tetzlaff JM, Akl EA, Brennan SE, Chou R, Glanville J, Grimshaw JM, Hróbjartsson A, Lalu MM, Li T, Loder EW, Mayo-Wilson E, McDonald S, McGuinness LA, Stewart LA, Thomas J, Tricco AC, Welch VA, Whiting P, Moher D (2021). The PRISMA 2020 statement: an updated guideline for reporting systematic reviews. BMJ.

[28] Norman CD, Skinner HA (2006). eHEALS: the eHealth literacy scale. J Med Internet Res.

[29] Cochrane handbook for systematic reviews of interventions Cochrane handbook for systematic reviews of interventions.

[30] Cochrane handbook for systematic reviews of interventions.

[31] Xie B (2011). Older adults, e-health literacy, and collaborative learning: an experimental study. J Am Soc Inf Sci Technol.

[32] Lee OEK, Kim DH (2019). Bridging the digital divide for older adults via intergenerational mentor-up. Res Soc Work Pract.

[33] Chiu CJ, Hu YH, Lin DC, Chang FY, Chang CS, Lai CF (2016). The attitudes, impact, and learning needs of older adults using apps on touchscreen mobile devices: results from a pilot study. Comput Hum Behav.

[34] Bevilacqua R, Strano S, Di Rosa M, Giammarchi C, Cerna KK, Mueller C, Maranesi E (2021). eHealth literacy: from theory to clinical application for digital health improvement. Results from the ACCESS training experience. Int J Environ Res Public Health.

[35] Chang SJ, Yang E, Lee KE, Ryu H (2021). Internet health information education for older adults: a pilot study. Geriatr Nurs.

[36] Nahm ES, Zhu S, Bellantoni M, Keldsen L, Russomanno V, Rietschel M, Majid T, Son H, Smith L (2019). The effects of a theory-based patient portal e-Learning program for older adults with chronic illnesses. Telemed J E Health.

[37] De Main AS, Xie B, Shiroma K, Yeh T, Davis N, Han X (2022). Assessing the effects of eHealth tutorials on older adults' eHealth literacy. J Appl Gerontol.

[38] Xie B (2011). Effects of an eHealth literacy intervention for older adults. J Med Internet Res.

[39] Nahm ES, Diblasi C, Gonzales E, Silver K, Zhu S, Sagherian K, Kongs K (2017). Patient-centered personal health record and portal implementation toolkit for ambulatory clinics: a feasibility study. Comput Inform Nurs.

[40] Jay GM, Willis SL (1992). Influence of direct computer experience on older adults' attitudes toward computers. J Gerontol.

[41] Nokes KM, Verkuilen J, Hickey DE, James-Borga JC, Shan J (2013). Developing a personal health record self-efficacy tool. Appl Nurs Res.

[42] Rotger JM, Cabré V (2022). Therapeutic alliance in online and face-to-face psychological treatment: comparative study. JMIR Ment Health.

[43] Jacobs RJ, Lou JQ, Ownby RL, Caballero J (2016). A systematic review of eHealth interventions to improve health literacy. Health Informatics J.

[44] Bandura A (1977). Self-efficacy: toward a unifying theory of behavioral change. Psychol Rev.

[45] Chang SJ, Choi S, Kim SA, Song M (2014). Intervention strategies based on information-motivation-behavioral skills model for health behavior change: a systematic review. Asian Nurs Res.

[46] Davis FD (1989). Perceived usefulness, perceived ease of use, and user acceptance of information technology. MIS Quarterly.

[47] Iqbal M, Zahidie A (2022). Diffusion of innovations: a guiding framework for public health. Scand J Public Health.

[48] Pourrazavi S, Kouzekanani K, Bazargan-Hejazi S, Shaghaghi A, Hashemiparast M, Fathifar Z, Allahverdipour H (2020). Theory-based E-health literacy interventions in older adults: a systematic review. Arch Public Health.

[49] Woodward AT, Freddolino PP, Blaschke-Thompson CM, Wishart DJ, Bakk L, Kobayashi R, Tupper C (2010). Technology and aging project: training outcomes and efficacy from a randomized field trial. Ageing Int.

[50] Xie B (2012). Improving older adults' e-Health literacy through computer training using NIH online resources. Libr Inf Sci Res.

[51] Xie L, Zhang S, Xin M, Zhu M, Lu W, Mo PKH (2022). Electronic health literacy and health-related outcomes among older adults: a systematic review. Prev Med.

[52] Chu A, Mastel-Smith B (2010). The outcomes of anxiety, confidence, and self-efficacy with internet health information retrieval in older adults: a pilot study. Comput Inform Nurs.

[53] Wild KV, Mattek NC, Maxwell SA, Dodge HH, Jimison HB, Kaye JA (2012). Computer-related self-efficacy and anxiety in older adults with and without mild cognitive impairment. Alzheimers Dement.

[54] Shiferaw KB, Tilahun BC, Endehabtu BF, Gullslett MK, Mengiste SA (2020). e-Health literacy and associated factors among chronic patients in a low-income country: a cross-sectional survey. BMC Med Inform Decis Mak.

[55] Cleary A, Dowling M (2009). Knowledge and attitudes of mental health professionals in Ireland to the concept of recovery in mental health: a questionnaire survey. J Psychiatr Ment Health Nurs.

[56] Fink A, Beck JC (2015). Developing and evaluating a website to guide older adults in their health information searches: a mixed-methods approach. J Appl Gerontol.

