# Supplementary material for: Effectiveness of Digital Health Literacy Interventions in Older Adults: Single-Arm Meta-Analysis
Source: J Med Internet Res. 2023 Jun 28;25:e48166. doi: 10.2196/48166 (PMC10365623; doi:10.2196/48166)

## Multimedia Appendix 1

## PubMed

(("Computer Literacy"[Mesh]) OR ((((((((((((Computer Literacy[Title/Abstract]) OR (Computer Literacies[Title/Abstract])) OR (Literacies, Computer[Title/Abstract])) OR (Literacy, Computer[Title/Abstract])) OR (ehealth literacy[Title/Abstract])) OR (e-health literacy[Title/Abstract])) OR (digital literacy[Title/Abstract])) OR (digital health literacy[Title/Abstract])) OR (mhealth literacy[Title/Abstract])) OR (m-health literacy[Title/Abstract])) OR (telehealth literacy[Title/Abstract])) OR (tele-health literacy[Title/Abstract]))) AND ((((((aged[Title/Abstract]) OR (elderly[Title/Abstract])) OR (old adult[Title/Abstract])) OR (older adult[Title/Abstract])) OR (old people[Title/Abstract])) OR (older people[Title/Abstract]))


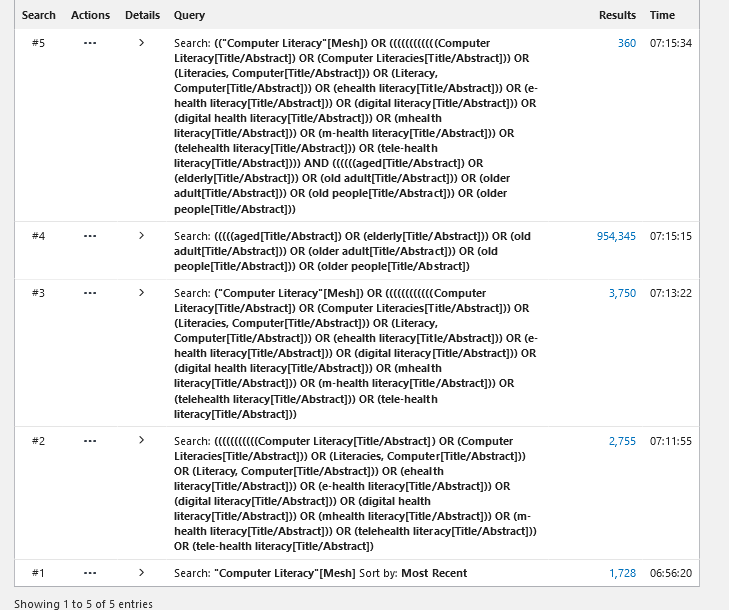


## Embase

#3. #1 AND #2

#2. aged:ab,ti OR elderly:ab,ti OR 'old adult':ab,ti OR 'older adult':ab,ti OR 'old people':ab,ti OR 'older people':ab,ti

#1. 'computer literacy':ab,ti OR 'computer literacies':ab,ti OR 'literacies, computer':ab,ti OR 'literacy, computer':ab,ti OR 'ehealth literacy':ab,ti OR 'e-health literacy':ab,ti OR 'digital literacy':ab,ti OR 'digital health literacy':ab,ti OR 'mhealth literacy':ab,ti OR 'm-health literacy':ab,ti OR 'telehealth literacy':ab,ti OR 'tele-health literacy':ab,ti


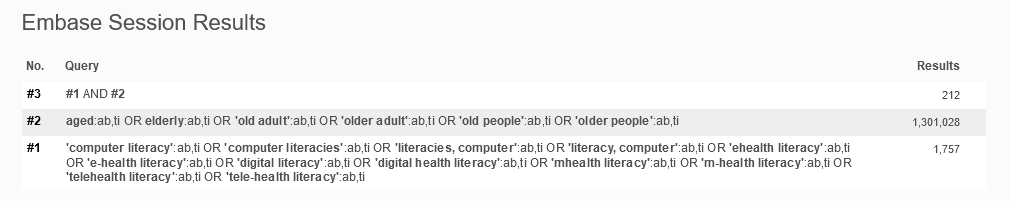


## Cochrane library

#1 MeSH descriptor: [Computer Literacy] explode all trees

#2 (Computer Literacy or Computer Literacies or Literacies, Computer or Literacy, Computer or ehealth literacy or e-health literacy or digital literacy or digital health literacy or mhealth literacy or m-health literacy or telehealth literacy or tele-health literacy):ti,ab,kw (Word variations have been searched)

#3 #1 or #2

#4 (aged or elderly or old adult or older adult or old people or older people):ti,ab,kw (Word variations have been searched)

#5 #3 and #4


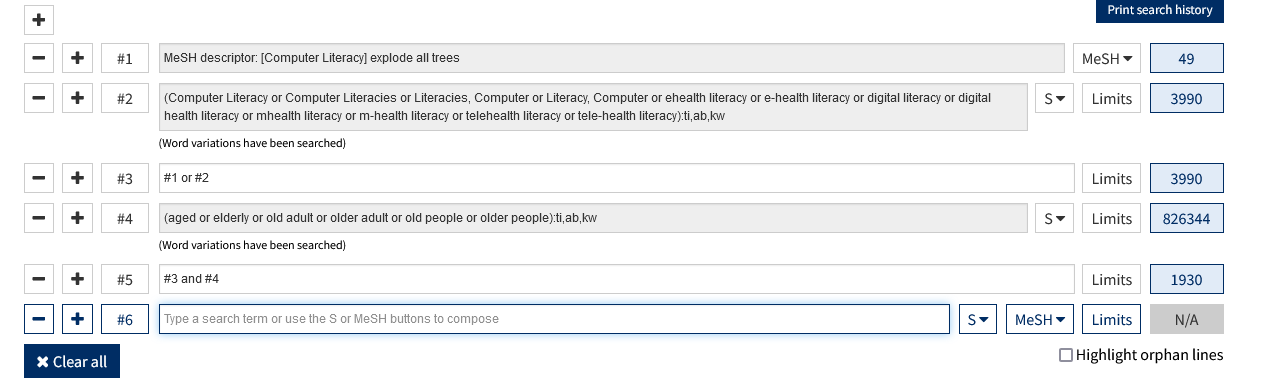


## Web of science

1: ((((((((((((TS=(Computer Literacy)) OR AB=(Computer Literacy)) OR AB=(Computer Literacies)) OR AB=(Literacies, Computer)) OR AB=(Literacy, Computer)) OR AB=(ehealth literacy)) OR AB=(e-health literacy)) OR AB=(digital literacy)) OR AB=(digital health literacy)) OR AB=(mhealth literacy)) OR AB=(m-health literacy)) OR AB=(telehealth literacy)) OR AB=(tele-health literacy)

2: (((((AB=(aged)) OR AB=(elderly)) OR AB=(old adult)) OR AB=(older adult)) OR AB=(old people)) OR AB=(older people)

3: #1 AND #2


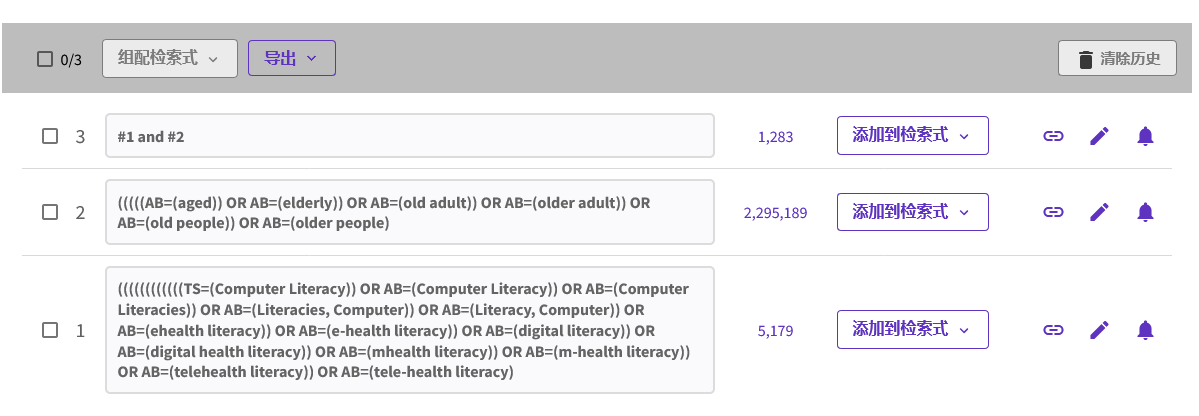

Supplement: Multimedia Appendix 1 [file jmir_v25i1e48166_app1.docx]
