# Supplementary material for: Effectiveness of Digital Health Literacy Interventions in Older Adults: Single-Arm Meta-Analysis
Source: J Med Internet Res. 2023 Jun 28;25:e48166. doi: 10.2196/48166 (PMC10365623; doi:10.2196/48166)
Supplement: Multimedia Appendix 2 [file jmir_v25i1e48166_app2.doc]

**Appendix 2: eHealth Literacy Scale**

**I would like to ask you for your opinion and about your experience using the Internet for health information. For each statement, tell me which response best reflects your opinion and experience *right now*.**

1. How **useful** do you feel the Internet is in helping you in making decisions about your health?

| 1 | 2 | 3 | 4 | 5 |
| --- | --- | --- | --- | --- |
| Not useful at all | Not useful | Unsure | Useful | Very Useful |

2. How **important** is it for you to be able to access health resources on the Internet?

| 1 | 2 | 3 | 4 | 5 |
| --- | --- | --- | --- | --- |
| Not important at all | Not important | Unsure | Important | Very important |

3. I know **what** health resources are available on the Internet

1)  Strongly Disagree

2)  Disagree

3)  Undecided

4)  Agree

5)  Strongly Agree

4. I know **where** to find helpful health resources on the Internet

1)  Strongly Disagree

2)  Disagree

3)  Undecided

4)  Agree

5)  Strongly Agree

5. I know **how** to find helpful health resources on the Internet

1)  Strongly Disagree

2)  Disagree

3)  Undecided

4)  Agree

5)  Strongly Agree

6. I know **how to use** the Internet to answer my questions about health

1)  Strongly Disagree

2)  Disagree

3)  Undecided

4)  Agree

5)  Strongly Agree

7. I know how to use **the health information** I find on the Internet to help me

1)  Strongly Disagree

2)  Disagree

3)  Undecided

4)  Agree

5)  Strongly Agree

8. I have the skills I need to **evaluate** the health resources I find on the Internet

1)  Strongly Disagree

2)  Disagree

3)  Undecided

4)  Agree

5)  Strongly Agree

9. I can tell **high quality** health resources from **low quality** health resources on the Internet

1)  Strongly Disagree

2)  Disagree

3)  Undecided

4)  Agree

5)  Strongly Agree

10. I feel **confident** in using information from the Internet to make health decisions

1)  Strongly Disagree

2)  Disagree

3)  Undecided

4)  Agree

5)  Strongly Agree

***Thank you!***

** Note: Questions #1 and #2 are recommended as supplementary items for use with the eHEALS to understand consumer’s interest in using eHealth in general. These items are not a formal part of the eHealth Literacy scale, which comprises questions #3-10.*
