# Supplementary material for: Effectiveness of Digital Health Literacy Interventions in Older Adults: Single-Arm Meta-Analysis
Source: J Med Internet Res. 2023 Jun 28;25:e48166. doi: 10.2196/48166 (PMC10365623; doi:10.2196/48166)
Supplement: Multimedia Appendix 3 [file jmir_v25i1e48166_app3.docx]

| **Reference, country** | **Design** | **Population** | **Intervention/Control methods** | **Theory framework** | **Duration** | **Results** |
| --- | --- | --- | --- | --- | --- | --- |
| Xie [31].  United States | quasi-experimental | 172 older adults with an average age of 70.4 | Collaborative group learning sessions in an informal learning environment (public libraries) | Self-efficacy theory | 4 weeks | Knowledge and skills of website use, and e-health literacy self-efficacy increased |
| Chiu et al [33].  Taiwan, China | quasi-experimental | 39 older adults with an average age of 69.5 | An 8-week training program on the theory and use of the APP | Technology Acceptance Model (TAM) and the Diffusion of Innovations model (DOI) | 8 weeks | Computer anxiety decreased and elderly e-health literacy efficacy increased |
| Lee et al [32].  United States | quasi-experimental | 59 older adults with an average age of 73.82 | Intergenerational Mentor-Up; offering educational opportunities for college students to interact with older adults |  | 2 weeks | Elderly eHealth literacy, technophobia, self-efficacy, and interest in technology increased, anxiety decreased and confidence boosted |
| Bevilacqua et al [34].  Italy | quasi-experimental | 58 older adults with an average age of 68.2 | Innovative eHealth training program: ACCESS  Raising awareness of eHealth and health literacy  Practicing new skills  Social communication  Self-evaluation and sustainability of the improvement |  | 4 weeks | The eHealth Literacy Scale(e-HEALS) mean value and satisfaction with the Survey of Technology Use (SOTU) increased |
| Chang et al [35].  Korea | quasi-experimental | 11 older adults aged 65 and older | Internet Health Information Education Project:  Computer basics  Understanding of the NHIP website  Use of the NHIP website  Use of the NAVER portal  Evaluating the credibility of online health information | The Information-Motivation- Behavioral skills (IBM) model | 5 weeks | Computer/Web knowledge, attitude toward internet-based health information, eHealth literacy score, searching performance scores, and level of understanding of internet-based health information showed significant improvement immediately after the intervention |
| Nahm et al [36].  United States | RCT | 272 older adults with an average age of 70 | IG: A theory-based patient portal e-learning program(T-PeP) for older people to use PPs to manage their health  CG: No specific intervention | Self-efficacy theory | 3 weeks | Patient portal knowledge, self-efficacy, health decision making and patient-provider communication improved |
| De Main et al [37].  United States | RCT | 99 older adults with an average age of 73.09 | IG: Multimedia learning: Using Online Tutorial Overlay Presenter (OnTOP) to recognize elements on a website and display visual annotations in an overlay on top of those elements  CG: Paper-based tutorial developed by the National Institute on Ageing (NIA) |  | 2 weeks | Knowledge about computer/Internet terms, eHealth literacy efficacy, knowledge about the quality of health information websites, and procedural skills in computer/Internet used improved significantly from pre- to posttest. |

**Note: RCT: randomized controlled trail; IG: intervention group; CG: control group**
